# Ultraviolet Treatment of Titanium to Enhance Adhesion and Retention of Oral Mucosa Connective Tissue and Fibroblasts

**DOI:** 10.3390/ijms222212396

**Published:** 2021-11-17

**Authors:** Takayuki Ikeda, Takeshi Ueno, Juri Saruta, Makoto Hirota, Wonhee Park, Takahiro Ogawa

**Affiliations:** 1 Weintraub Center for Reconstructive Biotechnology, Division of Advanced Prosthodontics, UCLA School of Dentistry, Los Angeles, CA 90095-1668, USA; ikeda.takayuki@nihon-u.ac.jp (T.I.); saruta@kdu.ac.jp (J.S.); mhirota@yokohama-cu.ac.jp (M.H.); drwon69@gmail.com (W.P.); togawa@dentistry.ucla.edu (T.O.); 2 Department of Complete Denture Prosthodontics, Nihon University School of Dentistry, 1-8-13 Kanda Surugadai, Chiyoda-ku, Tokyo 101-8310, Japan; 3 Advanced Prosthodontics, Graduate School of Medical and Dental Sciences, Tokyo Medical and Dental University, 1-5-45 Yushima, Bunkyo-ku, Tokyo 113-8549, Japan; 4 Department of Education Planning, School of Dentistry, Kanagawa Dental University, 82 Inaoka, Yokosuka 238-8580, Japan; 5 Department of Oral and Maxillofacial Surgery/Orthodontics, Yokohama City University Medical Center, 4-57 Urafune-cho, Minami-ku, Yokohama 232-0024, Japan; 6 Department of Dentistry, College of Medicine, Hanyang University, 222 Wangsimni-ro, Seongdong-gu, Seoul 04763, Korea

**Keywords:** connective tissue, fibroblast, surface characteristics, titanium implant, UV treatment

## Abstract

Peri-implantitis is an unsolved but critical problem with dental implants. It is postulated that creating a seal of gingival soft tissue around the implant neck is key to preventing peri-implantitis. The objective of this study was to determine the effect of UV surface treatment of titanium disks on the adhesion strength and retention time of oral connective tissues as well as on the adherence of mucosal fibroblasts. Titanium disks with a smooth machined surface were prepared and treated with UV light for 15 min. Keratinized mucosal tissue sections (3 × 3 mm) from rat palates were incubated for 24 h on the titanium disks. The adhered tissue sections were then mechanically detached by agitating the culture dishes. The tissue sections remained adherent for significantly longer (15.5 h) on the UV-treated disks than on the untreated control disks (7.5 h). A total of 94% of the tissue sections were adherent for 5 h or longer on the UV-treated disks, whereas only 50% of the sections remained on the control disks for 5 h. The adhesion strength of the tissue sections to the titanium disks, as measured by tensile testing, was six times greater after UV treatment. In the culture studies, mucosal fibroblasts extracted from rat palates were attached to titanium disks by incubating for 24, 48, or 96 h. The number of attached cells was consistently 15–30% greater on the UV-treated disks than on the control disks. The cells were then subjected to mechanical or chemical (trypsinization) detachment. After mechanical detachment, the residual cell rates on the UV-treated surfaces after 24 and 48 h of incubation were 35% and 25% higher, respectively, than those on the control surfaces. The remaining rate after chemical detachment was 74% on the control surface and 88% on the UV-treated surface for the cells cultured for 48 h. These trends were also confirmed in mouse embryonic fibroblasts, with an intense expression of vinculin, a focal adhesion protein, on the UV-treated disks even after detachment. The UV-treated titanium was superhydrophilic, whereas the control titanium was hydrophobic. X-ray photoelectron spectroscopy (XPS) chemical analysis revealed that the amount of carbon at the surface was significantly reduced after UV treatment, while the amount of TiOH molecules was increased. These ex vivo and in vitro results indicate that the UV treatment of titanium increases the adhesion and retention of oral mucosa connective tissue as a result of increased resistance of constituent fibroblasts against exogenous detachment, both mechanically and chemically, as well as UV-induced physicochemical changes of the titanium surface.

## 1. Introduction

The primary requirement for successful dental implant treatments is sufficient bone–implant integration [1,2,3,4,5,6,7,8,9,10,11]. This has led to a significant body of research on improving the integration between the surface of titanium implants and bone [2,4,5,12,13,14,15,16,17,18,19,20,21,22,23,24,25,26,27,28,29,30,31,32,33,34,35,36,37,38,39,40,41]. However, compatibility between the connective tissue and the implant neck region facing the gingiva is also required to facilitate wound healing and soft tissue sealing [42,43,44,45,46,47]. When the soft tissues do not attach to the implant quickly and tightly, bacterial infections can occur in the soft tissues around the implant neck region, particularly in the connective tissue, which can lead to peri-implant diseases [48,49]. Peri-implant diseases have a relatively high and increasing prevalence. One cross-sectional study reported that out of 211 participating individuals with dental implants, only 1.9% presented good peri-implant health, 3.8% presented clinical stability, 54.5% had mucositis, 39.8% had peri-implantitis, and 17.1% had severe peri-implantitis [43]. Another recent study from a university clinic database revealed that the prevalence of peri-implant diseases was 56.5% at the patient level and 27.9% at the implant level [46]. Furthermore, in patients with comorbidities such as diabetes, the risk of peri-implantitis increases. A meta-analysis reported that the risk of peri-implantitis for patients with diabetes is 50% higher compared to that in non-diabetic patients [44]. Therefore, there is a need for implant surface treatment technologies that can improve the affinity of the implant to connective tissues as well as to bone [31].

Ultraviolet (UV) treatment, known as UV photofunctionalization or UV activation, has been reported to change the physicochemical properties of the surface of titanium implants [50,51,52,53]. Biocompatibility depends on the physicochemical properties of the titanium surface, including the surface topography, wettability, and chemical composition [54,55,56,57,58,59,60]. UV treatment eliminates deposited hydrocarbons and increases the wettability of the titanium surface. This surface modification is crucial for improving cellular compatibility, bone formation, and clinical outcomes [50,61,62,63,64,65,66,67,68,69,70,71,72,73,74,75,76,77,78,79,80,81,82,83,84,85,86,87,88,89,90,91,92]. According to the results of past studies, osteoblast attachment to chemically cleaned and superhydrophilic titanium surfaces is enhanced following UV treatment, facilitating the spread, attachment, proliferation, and differentiation of cells [50,61,62,63,93,94,95,96,97,98,99,100,101].

Fibroblasts play an essential role in the production and remodeling of connective tissues, which is crucial for forming a peri-implant barrier [102]. To date, the effect of UV treatment on the biocompatibility of the titanium surface with fibroblastic cells has rarely been examined. In particular, soft tissue affinity, such as the adhesion and retention of connective tissues on UV-treated implant surfaces, has never been investigated. There are reportedly some differences in the cellular behavior of osteoblasts and fibroblasts [20]. For instance, the proliferation rate of osteoblasts increases with increasing surface roughness, whereas that of fibroblasts decreases with increasing roughness [20,103]. As the enhanced affinity of osteoblasts to UV-treated implant surfaces is promising for the success of dental implants, an analysis of fibroblast activity is of interest. Accordingly, we evaluated the ex vivo adherence strength and retention time of oral connective tissues as well as the in vitro adherence of oral mucosal fibroblasts on UV-treated and untreated (control) titanium surfaces. Specifically, we tested the hypothesis that the UV surface treatment of titanium implants would increase the mucosa connective tissue adhesion strength and resistance against detachment as well as the degree of fibroblast attachment and retention during mechanical and chemical stimulation.

## 2. Results

### 2.1. Surface Properties

**Figure 1:**
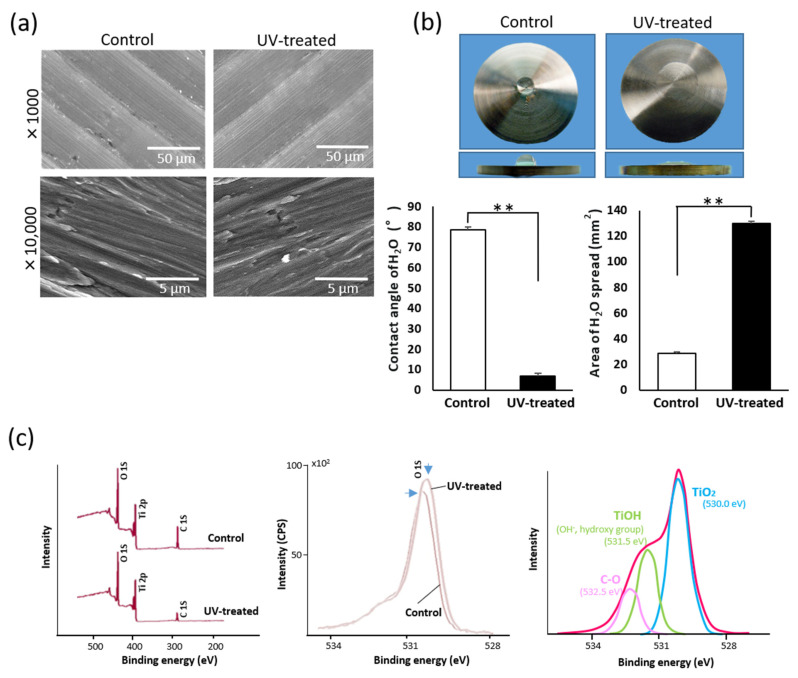
Surface characterization of machined titanium surfaces with and without UV treatment. (**a**) SEM images of four-week-old, machined titanium surfaces with and without UV treatment. (**b**) Hydrophilicity change after UV treatment. Optical images of 10 μL H_2_O droplets pipetted onto titanium surfaces (20 mm in diameter). The histograms show the contact angle and area of 10 μL H_2_O droplets. Data are mean ± SD (*n* = 3). ** *p* < 0.01, statistically significant difference between control and UV-treated surfaces. (**c**) XPS spectra for the untreated control and UV-treated titanium surfaces. The red arrowhead represents the C 1s peak showing a significant difference between the two surfaces (left panel). Comparison of O 1s peaks between the control and UV-treated titanium surfaces (middle panel). Three-peak detailed analysis applied to O 1s peaks.

The UV treatment had no obvious effect on the surface structure of the titanium disks (Figure 1a), indicating that UV light irradiation did not affect the surface topography; however, the surface wettability was drastically changed. The control disks stored for 4 weeks showed hydrophobicity, with a water contact angle (10 µL droplets) of more than 75° (Figure 1b). In contrast, the disks subjected to UV treatment were hydrophilic, with a water contact angle of 8°. The water droplets on the UV-treated titanium disks also spread to an area four times larger than on the control (Figure 1b).

X-ray photoelectron spectroscopy (XPS) survey scans on both the control and UV-treated surfaces generated typical spectra with elemental peaks of oxygen, titanium, and carbon (Figure 1c). A clear difference was observed in the intensity of the carbon (C 1s) peak between the two surfaces (left panel of Figure 1c). The calculated atomic percentages of carbon for the control and UV-treated surfaces were 47.3% and 28.2%, respectively. Furthermore, precision-superimposed spectra revealed a difference in the position of the O 1s elemental peak between the two surfaces (middle panel of Figure 1c). Therefore, we performed a detailed scan focusing on the O 1s region and divided the peak into three known sub-peaks of TiO_2_, TiOH, and C–O. Among these sub-peaks, the intensity of the C–O peak was smaller for the UV-treated surface (4.7%) than for the control surface (6.8%), as anticipated. Interestingly, the TiOH content increased from 14.0 to 17.5 at% after UV treatment.

### 2.2. Time to Detachment during Agitation

**Figure 2:**
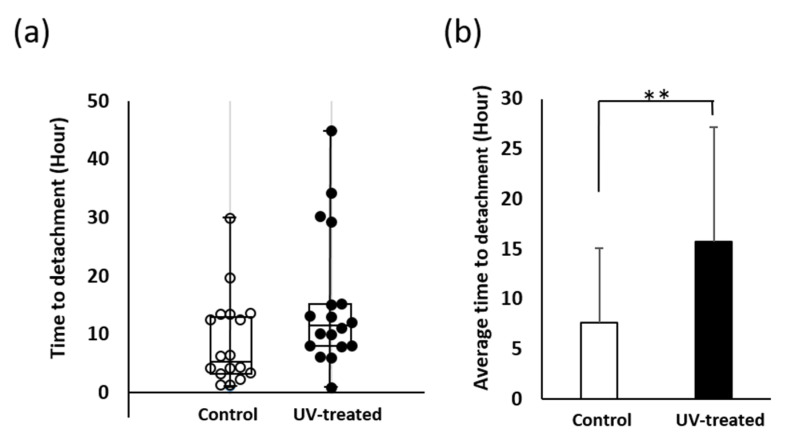
Time to detachment of mucosa connective tissues from titanium surfaces. (**a**) Box-and-whisker plots showing the distribution of time to detachment of tissue sections with and without UV treatment. (**b**) Comparison of average time to detachment of tissue sections with and without UV treatment. Data are mean ± SD (*n* = 18). ** *p* < 0.01, statistically significant difference between the control and UV-treated surfaces.

Ex vivo tests were conducted using sections of keratinized mucosa tissue to evaluate the time to detachment of cells on the titanium surface under agitation (Figure 2a). The average time to detachment was more than twice as long on the UV-treated surface (15.5 h) as that on the control surface (7.5 h) (Figure 2b). In addition, nine tissue sections (50%) detached from the control surface in less than 5 h, whereas only one section (5.6%) detached from the UV-treated surface in the same timeframe (Figure 2a). Conversely, the number of sections that remained attached for long periods was far higher for the UV-treated surface (10 h: twelve sections, 66.7%; 20 h: four sections, 22.2%) than for the control surface (10 h: seven sections, 38.9%; 20 h: one section, 5.6%) (Figure 2a). Although the time to detachment varied widely among specimens of the same groups, the data were more stable for tissue sections on UV-treated surfaces; the coefficient of variation was 0.98 for the control surface and 0.73 for the UV-treated surface.

### 2.3. Mucosa Connective Tissue Adhesion Strength

**Figure 3:**
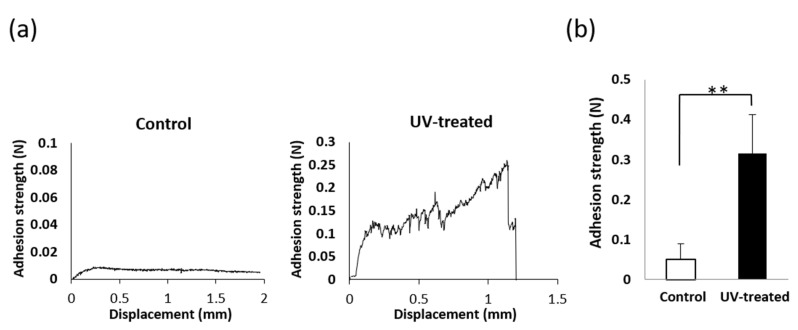
(**a**) Load–displacement curve obtained during pulling of mucosa connective tissue until detachment on control disk and UV-treated disk. The maximum load for the control disk and the break point for the UV-treated disk were recorded as adhesion strength. (**b**) Adhesion strength of mucosa connective tissue. Data are mean ± SD (*n* = 10). ** *p* < 0.01, statistically significant difference between the control and UV-treated surfaces.

The adhesion strength of mucosa tissue sections to the titanium surface was measured by tensile testing (Figure 3). A representative load–displacement curve is shown in Figure 3a. For the UV-treated disks, a clear drop-point was observed in the curves, whereas the drop-point tended to be unclear near the baseline for the control disks. In such cases, the maximum load was taken as the adhesion strength. The mucosa connective tissue adhesion strength on the UV-treated surface was nearly six times greater than that on the control surface (Figure 3b).

### 2.4. Fibroblast Attachment

**Figure 4:**
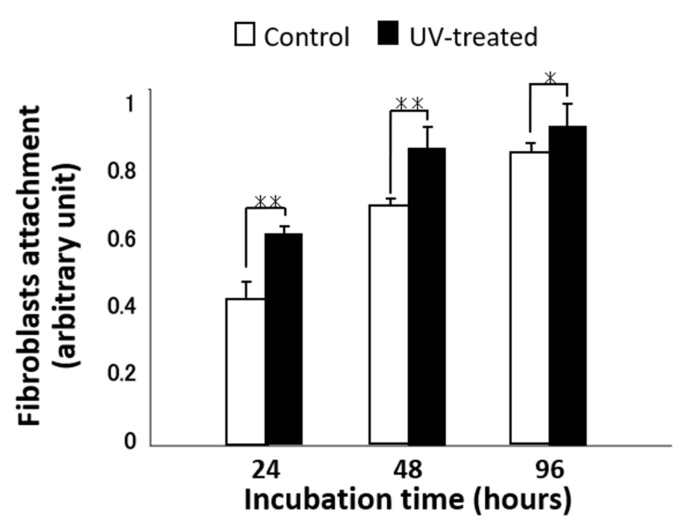
Total number of attached fibroblasts cultured for 24, 48, and 96 h on titanium disks with and without UV treatment. Data are mean ± SD (*n* = 6). ** *p* < 0.01, * *p* < 0.05, statistically significant difference between the control and UV-treated surfaces.

The total number of fibroblasts attached to the titanium surface was evaluated after incubation for 24, 48, and 96 h (Figure 4). After 24 h of incubation, the cell attachment level was 40% higher on the UV-treated surface than on the control surface. Although the number of attached cells on the UV-treated surface was significantly higher than that on the control surface at all time points, the difference gradually decreased with culture time; after 48 h, the UV-treated surface had a 25% higher attachment of fibroblasts compared to that on the control surface, while after 96 h, the difference was only 15%.

### 2.5. Remaining Fibroblasts after Mechanical Detachment

**Figure 5:**
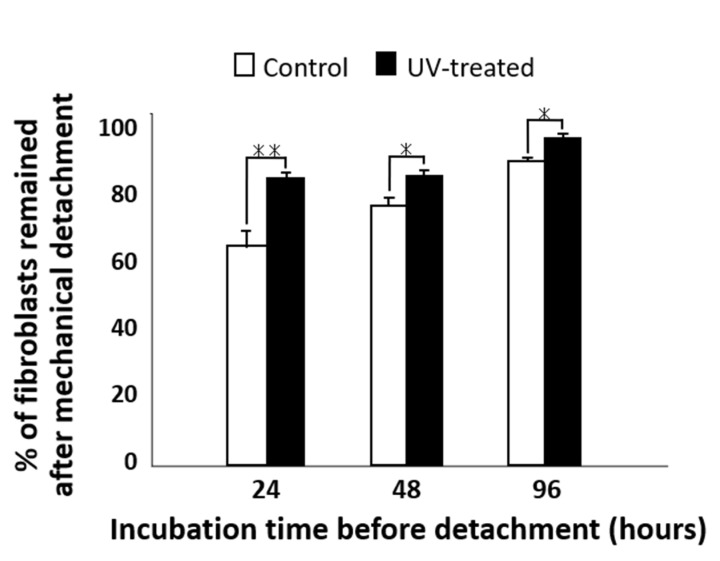
Percentage of attached fibroblasts remaining after mechanical detachment; cells were cultured for 24, 48, and 96 h on titanium disks with and without UV treatment. Data are mean ± SD (*n* = 6). ** *p* < 0.01, * *p* < 0.05, statistically significant difference between the control and UV-treated surfaces.

The percentage of fibroblast cells remaining after mechanical detachment was evaluated for both the control and UV-treated surfaces (Figure 5). After mechanical detachment, the residual cell rates on the UV-treated surfaces after 24 and 48 h of incubation were 35% and 25% higher, respectively, than those on the control surfaces. The difference in the residual cell rate between the UV-treated and control surfaces gradually decreased with time. After 96 h of incubation, more than 90% of cells remained after mechanical detachment on both the control and UV-treated surfaces, with a difference of 5% between the two surfaces.

### 2.6. Remaining Fibroblasts after Chemical Detachment by Trypsin

**Figure 6:**
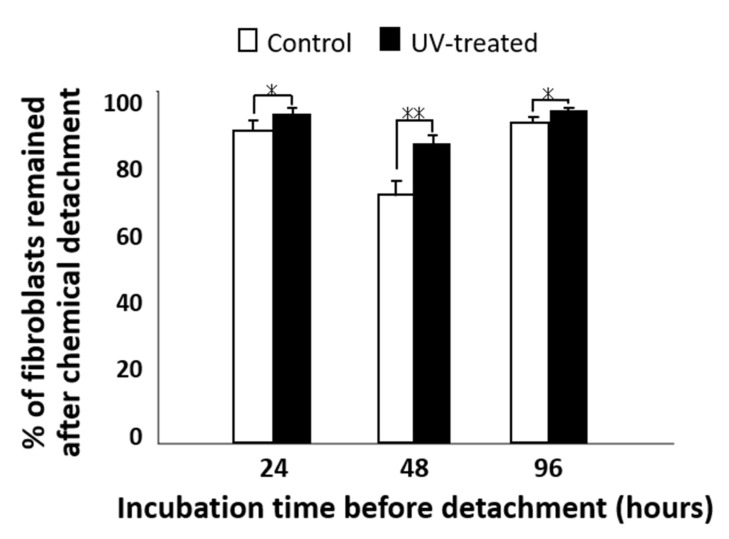
Percentage of attached fibroblasts remaining after chemical detachment; cells were cultured for 24, 48, and 96 h on titanium disks with and without UV treatment. Data are mean ± SD (*n* = 6). ** *p* < 0.01, * *p* < 0.05, statistically significant difference between the control and UV-treated surfaces.

Next, the percentage of remaining cells after chemical detachment was evaluated for both the control and UV-treated surfaces (Figure 6). Chemical stimulation was applied by adding trypsin to the culture medium. Although more than 90% of the cells cultured for 24 and 96 h remained on both the control and UV-treated surfaces after trypsinization, there was a significant difference in the percentage of residual cells between the control and UV-treated surfaces. A comparatively high difference was detected for the cells cultured for 48 h, while the remaining rate after chemical detachment was 74% on the control surface and 88% on the UV-treated surface.

### 2.7. Validation of Vinculin Expression Using NIH3T3 Cells after Mechanical Detachment

**Figure 7:**
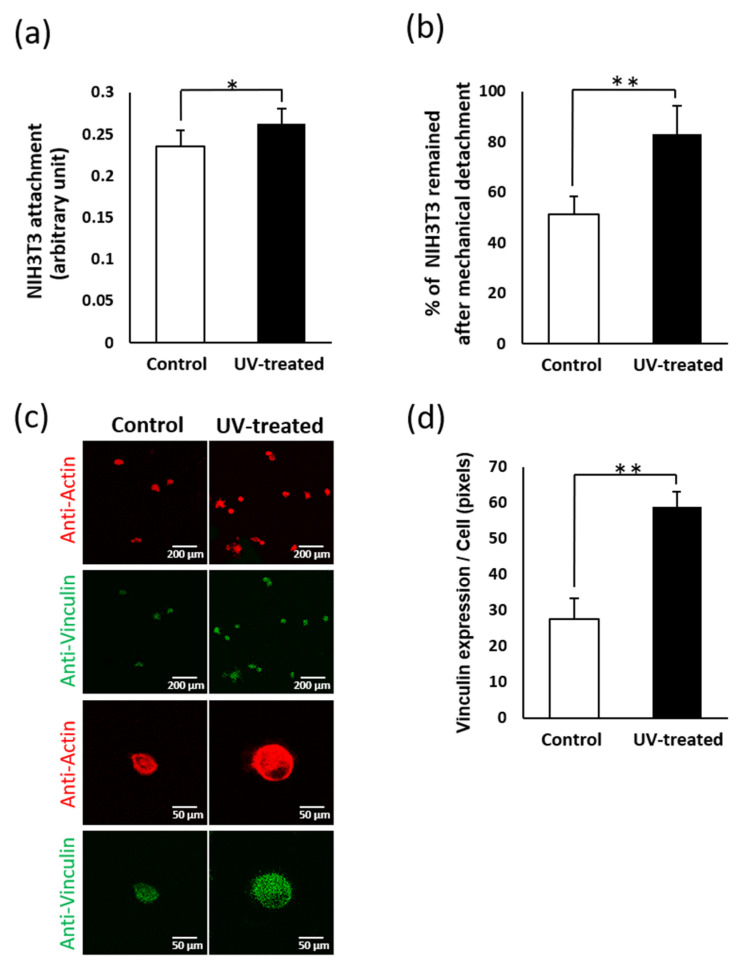
(**a**) Total number of attached NIH3T3 cells cultured for 24 h on titanium disks with and without UV treatment. Data are mean ± SD (*n* = 6). * *p* < 0.05, statistically significant difference between the control and UV-treated surfaces. (**b**) Percentage of attached NIH3T3 cells remaining after mechanical detachment; cells were cultured for 24 h on titanium disks with and without UV treatment. Data are mean ± SD (*n* = 6). ** *p* < 0.01, statistically significant difference between the control and UV-treated surfaces. (**c**) Cytoskeletal arrangement and expression of focal adhesion protein vinculin in NIH3T3 cells that remained on the titanium surface after mechanical detachment. Representative confocal microscopic images of cells stained with rhodamine phalloidin for actin filaments (red) and anti-vinculin (green). Cells cultured on titanium disks with and without UV treatment for 24 h were used. (**d**) Image-analysis-based vinculin expression. Data are mean ± SD (*n* = 6).

The expression of vinculin, a representative cell adhesion protein, was measured after the mechanical detachment of NIH3T3 cells after 24 h of incubation. The number of attached cells before detachment was 1.1 times higher on the UV-treated surface than on the control surface (Figure 7a), whereas the number of attached cells after detachment was 1.6 times higher on the UV-treated surface (Figure 7b). Vinculin expression was two times higher on the UV-treated surface than on the control surface and remained so even after mechanical detachment (Figure 7c).

## 3. Discussion

To the best of our knowledge, this study presents the first demonstration of mucosa connective tissue compatibility on UV-treated titanium surfaces through ex vivo mucosal adhesion experiments. This novel ex vivo test proves the positive effect of UV treatment on connective tissue and fibroblast affinity. As mentioned in the Introduction, several studies have demonstrated that osteoblastic activity is enhanced by UV treatment of titanium, but fibroblastic activity on UV-treated titanium has rarely been investigated. Herein we demonstrated that fibroblast attachment is 40% higher on UV-treated titanium than that on control titanium after 24 h of incubation, which is consistent with the trend reported for osteoblasts in previous studies [50,61,62].

Titanium absorbs organic impurities such as polycarbonyls and hydrocarbons from the atmosphere, water, and cleaning solutions [104,105]. The detection of high concentrations of carbon on the surface of titanium implants indicates that such contamination may be unavoidable [106,107]. In the present study, the XPS analysis demonstrated that the carbon on the control surface was reduced by UV treatment, which is consistent with the results of several previous studies [64,94]. UV light irradiation of titanium surfaces causes two chemical reactions, namely photolysis and photocatalysis, both of which decompose organic compounds. Photolysis is the direct decomposition of organic compounds by high-intensity light, while photocatalysis decomposes carbon compounds in the titanium dioxide passive layer by exciting electrons from the valence band to the conduction band, thus catalyzing the chemical reaction [108].

Previous reports have suggested a link between surface hydrocarbons and the hydrophilicity of titanium; the water contact angle has been found to increase with the absorption of hydrocarbons [109]. Oxygen species derived from O_2_ in air, which effectively increase hydrophilicity, are covered by adsorbed hydrocarbons. The intensity of the O 1s peak was lower for the control surface than that for the UV-treated surface. It is therefore likely that the wettability was reduced because of the adsorption of organic molecules on the surface of the untreated titanium disk. Generally, wettability is governed by the number of surface hydroxyl (OH) groups [110]. One study demonstrated that increasing the number of OH groups increases wettability as well as cell adhesion, protein adsorption, and cell attachment [111]. Another study reported that protein immobilization can be enhanced by increasing the number of OH groups on titanium dioxide surfaces [112]. In contrast, the masking of OH groups by carbon accumulation decreases the attachment of osteoblasts to titanium [113]. In addition, previous studies examining the effects of carbon contamination on titanium surfaces on bone formation have revealed a time-dependent increase in carbon content and decrease in wettability, with less bone forming around titanium implants that have been stored for four weeks prior to implantation than around newly manufactured implants; this phenomenon was named the “biological aging of titanium” [10,59]. Therefore, the removal of organic molecules is an important challenge in regenerating the biological capacity of implants. UV treatment could be one way to decompose impurities from the titanium surface, both directly and indirectly, and to induce simultaneous superhydrophilicity, resulting in the enhancement of biological reactions.

Clinically, the connective tissue barrier is very important for protecting against bacterial infections. A histological study reported that the fibroblast-rich barrier tissue that lies immediately next to the implant surface plays an important role in maintaining an adequate seal against the external environment [114]. In the peri-implant area, connective tissue is attached much more tightly than epithelial tissue to the titanium surface; thus, faster connective tissue formation is required before epithelial growth into the peri-implant sulcus. The fibroblast affinity assays in the present study demonstrate that UV treatment can improve connective tissue affinity and adhesion to the implant surface. The strength of cell attachment on the UV-treated surface was significantly higher than that on the control surface; however, the difference between the two surfaces gradually decreased over time. This indicates that although the initial speed of cell attachment is faster on UV-treated surfaces than that on control surfaces, cell proliferation on the control surface increases with time. Similarly, the retention rate of fibroblasts after mechanical detachment was greater on the UV-treated surface than on the control surface at the initial stage (first 24 h of incubation), and the difference in retention rate decreased with increasing culture time. This indicates that the settling down of the titanium surface and the maturation speed are accelerated by UV treatment.

As for chemical detachment, the percentage of cells remaining after exposure to trypsin was constantly higher on the UV-treated surface than that on the control surface, indicating that cell adhesion proteins are more resilient to chemical detachment after UV treatment. However, the rate of cell retention was lower after culturing for 48 h than that after 24 and 96 h, which complicates interpretation. It is possible that the timing of trypsin stimulation led to these differences; that is, the cellular sensitivity to proteolysis by trypsin may be different during cell proliferation and after cell adhesion. If cell proliferation activity was predominant at 48 h, this would explain why the cell detachment was increased compared to that at other times. The increase in fibroblast adhesion after UV treatment might be enhanced by the associated upregulation of vinculin, which is involved in the linkage between cell adhesion molecules, integrins, and actin filaments and plays a key role in initiating cell adhesion and cell shape formation [115,116,117,118]. The retention of fibroblasts in the present study was consistently over 80% on the UV-treated surfaces, regardless of the detachment method, suggesting that the initial difference in biological potential may determine the subsequent bioactivity of the titanium surface, potentially resulting in faster and stronger adhesion of connective tissues onto UV-treated implant surfaces.

There have been many trials to increase fibroblast attachment to titanium surfaces by using bioactive protein coatings such as fibroblast growth factor-2 [119], laminin [120], fibronectin [121,122], and collagen type-1 [123]. These techniques successfully increase fibroblast attachment, but the use of growth factors or cytokines should be well managed to maintain their activity, while coating techniques suffer from the propensity of detachment at the interface between the base material and coating. UV treatment of implants is an easy and simple choice for clinical applications. The most crucial and advantageous difference is that UV treatment does not require pre-coating or protein immobilization. UV-treated titanium can collect or attract host-derived proteins on its surface, which results in enhanced bioactivity for both osteoblasts and fibroblasts. In addition, the attachment of gingival epithelial cells is enhanced on UV-treated titanium surfaces [124]. Taken together with the present study, soft tissue sealing could be enhanced by UV treatment. Nevertheless, optimization is necessary to control the balance between epithelial and connective tissue formation. In addition, our study is limited in that it is difficult to evaluate the differences between groups after more than 96 h of culturing, as the cells reach high levels of confluence. Therefore, long-term evaluations of the effect of UV treatment on connective tissue sealing are required. Finally, further in vivo studies are required to identify the positive effects of UV-treated titanium implants and to provide a new strategy to promote soft tissue sealing and reduce the risk of peri-implantitis.

## 4. Materials and Methods

### 4.1. Titanium Disks and UV Treatment

Commercially pure titanium disks (20 mm diameter × 15 mm thickness) with machined surfaces (grade 2) were stored in dark, ambient, sterile conditions for 4 weeks according to a previously established protocol [63,65]. UV treatment was performed using a UV photo device (TheraBeam Affiny, Ushio Inc., Tokyo, Japan) for 15 min. The UV photo device comprised a rectangular chamber (180 mm length × 21 mm width × 90 mm height) with UV light sources on the right and left surfaces. The disks were centered between the light sources so that each light source was approximately 8.625 mm from the sides of the disks. The UV wavelengths were a combination of UVA, UVB, and UVC under a proprietary protocol.

The surface morphology and chemical composition of the titanium disks were evaluated using scanning electron microscopy (XL30, Philips, Eindhoven, Netherlands) and XPS (Axis Ultra DLD spectrometer, Kratos Analytical, Shimadzu, Kyoto, Japan), respectively, and the hydrophilicity was evaluated by measuring the contact angle and spread area of 10-microliter droplets of double-distilled water. The contact angle was measured using a contact angle meter (CA-X, Kyowa Interface Science, Tokyo, Japan) and the spread area was measured from photographs of the droplets using ImageJ (NIH, Bethesda, ML, USA).

A total of 194 titanium disks were used, including 97 UV-treated disks and 97 control disks. Of these, 18 were used for surface characterization analyses, 12 for tissue adhesion time measurements, 20 for tissue adhesion strength assays, 36 for fibroblast attachment studies, 36 for mechanical detachment studies, 36 for chemical detachment studies, and 36 for NIH3T3 cell adhesion assays. In all experiments, half the disks were UV-treated and the other half were control samples.

### 4.2. Keratinized Mucosa Connective Tissue Attachment

**Figure 8:**
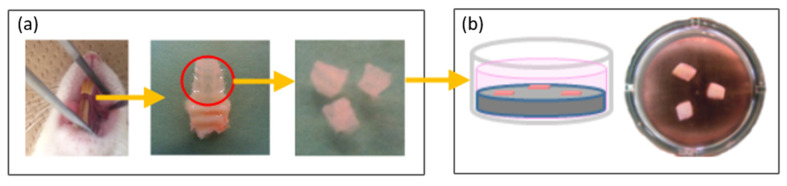
Experimental procedure for the setting of mucosa connective tissue. (**a**) Keratinized mucosa connective tissues derived from rat palate. Three to five sections (one section: 3 × 3 mm) were obtained from one palate. (**b**) Keratinized mucosa connective tissues setting on titanium surface in culture medium. Three sections were placed on each titanium disk and incubated for 24 h before the detachment test.

Keratinized mucosa connective tissues were collected from 8-week-old male Sprague Dawley rat palates. The collected palatal mucosae were carefully washed with phosphate-buffered saline (PBS) to remove tissues other than the mucosa connective tissue and shaped into tissue sections with dimensions of 3 × 3 mm. As three to five sections can be collected from each palate, the required sections were collected from 20 rats (Figure 8a). Three tissue sections were placed approximately equidistantly on each titanium disk, moistened with alpha-modified Eagle’s medium to prevent the tissue sections from drying out, and left for approximately 1 h. After confirming that the tissue sections were attached to the titanium disks, the disks were transferred to a culture dish and 1 mL of alpha-modified Eagle’s medium was gently added. Finally, tissue section attachment was established by 24-h incubation (Figure 8b).

### 4.3. Adhesion Time Measurement

To evaluate the retention force of the mucosa connective tissue sections on the titanium disks, we performed mucosa connective tissue section detachment assays. Titanium disks with adhered tissue sections were placed in 12-well culture plates. These culture plates were mechanically and continuously stimulated with a shaker (Slow Shaker, Corning Inc., Corning, NY, USA) at an agitating amplitude of 10 mm and a frequency of 30 Hz. Every 10 min, the tissue sections were observed, and those that had moved or floated from their original positions were recorded as having detached at the observation time. Six UV-treated and six control disks were used in this experiment.

### 4.4. Adhesion Strength Assay

The adhesion strengths of the tissue sections were evaluated by tensile testing. After 24 h of incubation to establish tissue attachment on the titanium disks, a hook was attached vertically to the center of the tissue section using superglue. As soon as the hook was fixed, it was pulled vertically upwards using a testing machine (Instron 5544 Electromechanical Testing System, Instron, Canton, MA, USA) at a crosshead speed of 0.05 mm/min. The retention strength was determined by measuring the peak of the load–displacement curve. Ten UV-treated and ten control disks were tested, with one tissue section selected for tensile testing per disk.

### 4.5. Fibroblasts and NIH3T3 Cell Culture

Keratinized mucosal fibroblasts were obtained from the explants of the oral palatal mucosa of 8-week-old male Sprague Dawley rats. Cells were grown in Dulbecco’s modified Eagle’s medium (DMEM; GIBCO BRL, Grand Island, NY, USA) supplemented with 10% fetal bovine serum (FBS) and 1% penicillin–streptomycin solution (Gibco BRL) under conditions of humidified 5% CO_2_/95% air at 37 °C. The medium was changed every 3 days, and the cells were passaged with trypsin–EDTA (GIBCO BRL, Grand Island, NY, USA) when they became confluent. All experiments were performed using early passaged cells. Mouse embryonic fibroblasts (NIH3T3, American Type Culture Collection, Manassas, VA, USA) were cultured in DMEM supplemented with 10% FBS, 2 mM L-glutamine, and 1% penicillin–streptomycin solution.

### 4.6. Cell Adhesion Assay

Fibroblast cell adhesion assays were performed to assess the cell retention force on the titanium surface. After incubation for 24, 48, or 96 h, the culture was rinsed twice with PBS and transferred to a new culture plate. For mechanical stimulation, the disks were agitated for 10 min (amplitude: 10 mm, frequency: 30 Hz) to detach the cells from the surface. For chemical stimulation, the disks were incubated in 1 mL of 0.025% trypsin solution for 10 min to detach the cells from the surface. The total number of cells attached to the titanium disks after static incubation for 10 min was counted. For each of the three assays, six UV-treated and six control disks were tested for each incubation time (total number of disks = 108). Cell counts were measured by colorimetry using WST-1 reagent (Roche Applied Science, Indianapolis, IN, USA). WST-1 reagent (100 µL) was added to the cultures at 37 °C for 1 h; then, the absorbance was measured in each well at a wavelength of 450 nm using a plate reader (Bio-Rad, Hercules, CA, USA). Cell retention (%) was calculated as [(remaining cells on disc after exfoliation)/(total number of cells attached to disks)] × 100.

NIH3T3 cells were subjected to cell adhesion tests similar to the above mechanical stimulation, and the number and morphology of the cells were evaluated for confirmation. Six UV-treated and six control disks were tested for each incubation time (total number of disks = 36). In addition, the expression level of the focal adhesive protein vinculin was measured. For staining, cells were fixed in 10% formalin for 8 min and then stained with the fluorescent dye rhodamine phalloidin (Invitrogen, Grand Island, NY, USA). To observe the intracellular expression and localization of vinculin, cells were additionally stained with rabbit anti-vinculin monoclonal antibodies (Abcam, Cambridge, UK) followed by FITC-conjugated anti-rabbit secondary antibodies (Abcam). The specimens were embedded in mounting medium (Vectashield, Fisher Scientific, Pittsburgh, PA, USA) and observed using a confocal laser scanning microscope (CLSM; Leica TCS-SP5 STED confocal multiphoton microscope, Leica Microsystems, Heidelberg, Germany). Vinculin expression was measured using ImageJ software (NIH, Bethesda, ML, USA). 

### 4.7. Statistical Analyses

The hydrophilicity was evaluated using three different titanium disks (*n* = 3). The adhesion time of tissue sections was evaluated using 18 sections for the control and UV-treated specimens (*n* = 18). Tensile tests were performed on 10 tissue sections for the control and UV-treated specimens (*n* = 10). Six samples were analyzed in all the cell culture experiments (*n* = 6). Welch’s t-test and Student’s t-test were used to examine the difference between the control and UV-treated groups at each time point, with *p*-values of <0.05 considered significant. Statistical analyses were performed using IBM SPSS Statistics version 20 (IBM, Armonk, NY, USA).

## 5. Conclusions

UV treatment of titanium enhanced the adhesion and retention of mucosa connective tissues and increased and accelerated fibroblast attachment and resistance against mechanical and chemical detachment. This connective tissue compatibility is based on UV-induced superhydrophilicity and the decomposition of carbon impurities. From a clinical perspective, UV treatment of titanium is a promising strategy for improving connective tissue sealing to prevent bacterial invasion of the peri-implant region.

## Data Availability

The data presented in this study are available on request from the corresponding author.
